# Hydatid Cyst of the Knee: A Case Report

**DOI:** 10.5812/kmp.iranjradiol.17351065.3162

**Published:** 2011-11-25

**Authors:** Hossein Ghanaati, Mehdi Mohammadifar, Mahsa Ghajarzadeh, Kavous Firouznia, Marzieh Motevalli, Amir Hossein Jalali

**Affiliations:** 1 Department of Radiology, Medical Imaging Center, Advanced Diagnostic and Interventional Radiology Research Center (ADIR), Imam Khomeini Hospital,Tehran University of Medical Sciences, Tehran, Iran; 2 Brain and Spinal Injury Repair Research Center (BASIR), Tehran University of Medical Sciences, Tehran, Iran; 3 Department of Radiology, Shahid Radjaee Cardiovascular Medical Center, Tehran University of Medical Sciences, Tehran, Iran

**Keywords:** Knee, Echinococcus, Infection

## Abstract

Hydatid disease is a parasitic infection which occurs in specific geographical areas such as the Mediterranean region. We report a case of hydatid cyst of the knee in a 34-year-old man who was admitted with inability to walk and a painful knee. He had a past history of liver infection nine years ago. Laboratory findings were negative. According to the high prevalence of hydatid disease in Iran, it should be considered as a differential diagnosis of Baker’s cyst, synovial cyst and lipoma.

## 1. Introduction

Hydatid disease is one of the endemic diseases of the Mediterranean region. It has been reported in 1% of Iranian patients admitted for surgery [1]. The larval form of Echinococcus, a tapeworm which exists in the small intestine of carnivores, is the causative agent of this disease. The most affected site is the liver (65-75%) followed by the lung (15-25%) and the kidney (3%). The musculoskeletal system is rarely affected (1-4%) [2][3]. In endemic regions, the disease should be considered as a differential diagnosis of lipoma, Baker’s cyst and synovial cyst [4]. We present a rare case of hydatid disease of the knee who developed hydatid cyst of the knee nine years after hydatid disease of the liver.

## 2. Case Presentation

A 34-year-old man from a rural area was admitted with increasing pain over the right knee for the past 6 months and inability to walk. At 25 years of age, he had been diagnosed with hydatid disease in the liver and was treated for one month with albendazole (10 mg/kg). On physical examination the right knee was swollen, hot and painful. Examination revealed limitation in knee movement (15°/70°). Table 1 shows the patient’s laboratory findings. Other serologic tests were negative.

**Table 1:** The Patient’s Laboratory Findings

	**Results**
ESR^a^, mm/h	18
Hemoglobin, g/dL	19
Leukocyte count, cells/mL	6700
Eosinophil count, cells/mL	800

^a^ Abbbreviation: ESR, Erythrocyte sedimentation rate

**Figure 1:**
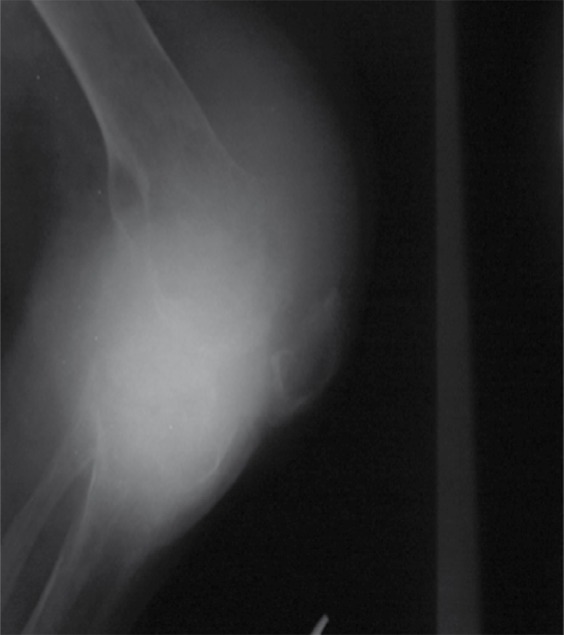
A 34-Year-Old Man Presenting With a Hot and Painful Right Knee With Severe Soft Tissue Swelling. Plain radiography shows multiple lytic, well-defined, narrow transitional zone bone lesions in the distal of the femur and proximal of the tibia with patellar involvement, juxta-articular osteoporosis, soft tissue density in the suprapatellar pouch.

Radiography of the right knee showed severe soft tissue swelling, decreased joint space, multiple lytic, well-defined, narrow transitional zone bone lesions in the distal of the femur and proximal of the tibia with patellar involvement, juxta-articular osteoporosis and soft tissue density in the suprapatellar pouch (Figure 1).

**Figure 2:**
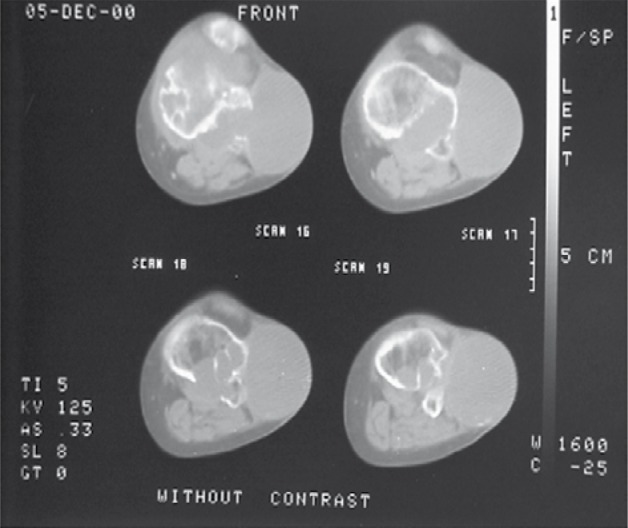
CT Scan Without Contrast in the Same Patient. It shows multiple destructive cystic lesions in the proximal of the tibia and distal of the femur which some of them show extra-osseous extension.

**Figure 3:**
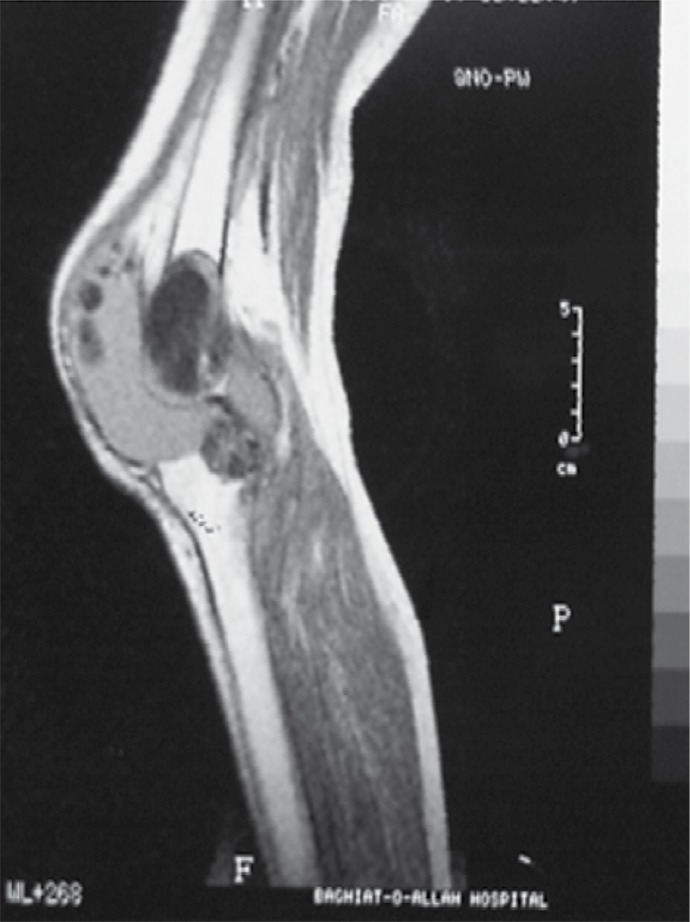
MRI (T1W-MRI) Without Contrast. It shows low signal intensity lesions in the proximal of the tibia and distal of the femur with joint space involvement.

Computed tomography (CT) showed multiple destructive cystic lesions in the proximal of the tibia and distal of the femur in which some of them extra-osseous extension was detected (Figure 2). In T1W-MRI, low signal intensity lesions are seen in the proximal of the tibia and the distal of the femur with joint space involvement (Figure 3). Fluid in the joint space is also noted. Cranial and thoracic CT scans and abdominal ultrasound were normal. Micro and macro histopathological examination confirmed the diagnosis of hydatid cyst and no evidence of recurrence was detected during the 2-year follow up.

## 3. Discussion

Hydatid disease of the musculoskeletal system is a rare condition (1-4%) against the high involvement of the other organs such as the liver, the lung and the kidney [2]. Most of the musculoskeletal hydatid diseases are secondary to liver, lung or subphrenic infections so symptoms may be presented within 5-20 years [5]. In our case, hydatid cyst of the knee was secondary to liver involvement after nine years.

Dudkiewicz et al. noted thigh muscle hydatidosis secondary to lung involvement in a 14-year-old boy three years after lung lobectomy [6]. Similar to our patient, their case lived in a village and he was symptom-free after the first hydatidosis treatment. Garcia-Alvarez et al. found 13 cases of paravertebral hydatidosis secondary to systemic infections [7].

Acids of the stomach break the eggs so the larva may be transported by the portal vein to the target organs, mostly the liver and the lungs. In the target organs, fluid-filled hydatid cysts which contain daughter cysts develop. According to the high blood flow of the right lobe of the liver, most of the cysts are formed in this site [8]. Rupture of the cyst is one of the most important complications of musculoskeletal hydatidosis that results in release of daughter cysts and extension of the disease sometimes leading to anaphylactic shock [9].

Laboratory findings and imaging modalities like sonography, CT, MRI and plain radiography may help the diagnosis of musculoskeletal hydatidosis [3] by showing the multilocular cysts and sometimes the scolices. These modalities should be used carefully to avoid cyst spread. Surgical treatment with careful surgical margins should be applied for musculoskeletal hydatidosis treatment. In 50% of hydatid diseases, serologic findings are negative like our case, although eosinophilia is revealed in nearly 25% of the affected cases [6].

In conclusion, in endemic regions such as Iran hydatid disease should be considered as the differential diagnosis of soft tissue masses [4].
